# Nutrition and Osteoporosis Prevention

**DOI:** 10.1007/s11914-024-00892-0

**Published:** 2024-09-25

**Authors:** René Rizzoli, Thierry Chevalley

**Affiliations:** Service of Bone Diseases, Geneva University Hospitals and Faculty of Medicine, 1211 Geneva 14, Switzerland

**Keywords:** Dietary intakes, Nutrients, Foods, Dietary patterns, Fracture, Protein, Dairies, Minerals, Gut microbiota

## Abstract

**Purpose of Review:**

Osteoporosis affects 50% of women and 20% of men after the age of 50. Fractures are associated with significant morbidity, increased mortality and altered quality of life. Lifestyle measures for fragility fracture prevention include good nutrition including adequate protein and calcium intakes, vitamin D sufficiency, and regular weight bearing physical exercise.

**Recent Findings:**

Dietary protein is one of the most important nutritional considerations as it affects bone mineral density, trabecular and cortical microstructure, and bone strength. When calcium intake is sufficient, higher dietary protein intake is associated with lower risk of fracture. Dairy products are a valuable source of calcium and high quality protein. Dairy product consumption, particularly fermented dairy products, are associated with a lower risk of hip fracture and vegan diets are associated with increased fracture risk. Other dietary factors associated with reduced fracture risk include at least 5 servings per day of fruits and vegetables, regular tea drinking, adherence to a Mediterranean diet and other dietary patterns which provide fibers, polyphenols and fermented dairy products. Such dietary patterns may confer health benefits through their effect on gut microbiota composition and/or function.

**Summary:**

A balanced diet including minerals, protein, fruits and vegetables is an important element in the prevention of osteoporosis and of fragility fracture.

## Introduction

**Fig. 1:**
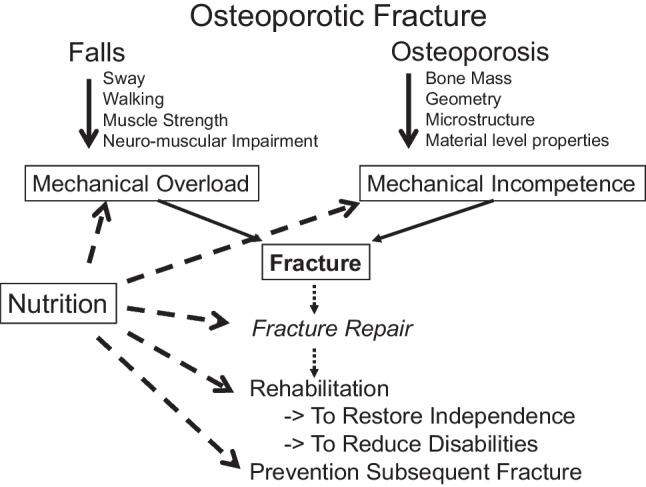
Determinants of fracture risk. Mechanical overload and mechanical incompetence, as well as fracture healing and secondary fracture prevention are influenced by nutritional intakes

**Fig. 2:**
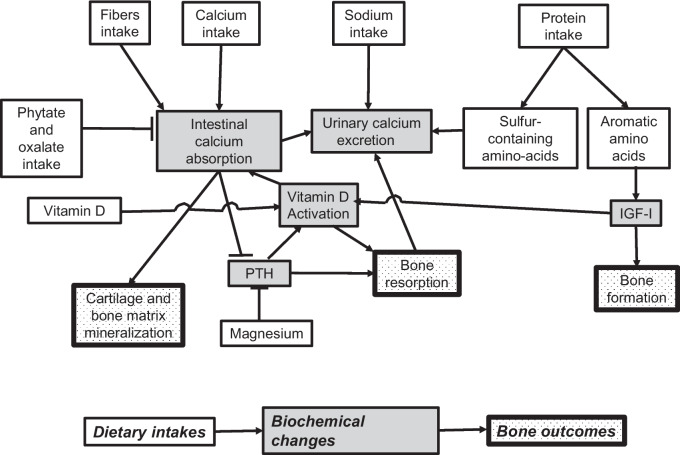
Nutrient intake, main physiological responses and bone health variables. Nutrients are in white, biochemical changes in response to nutrients intake are in gray and bone related outcomes are in stipple. Arrows indicate stimulation and flat arrowheads indicate inhibition. Adapted from [2] with permission from the publisher

Osteoporotic fracture risk increases as bone mass decreases and bone microstructure deteriorates following peak bone mass attainment, with an accelerated bone loss occurring after menopause [1]. The lifetime risk of sustaining an osteoporotic fracture is approximately 1 out of 2 women and 1 out of 5 men by the age of 50 years over the remaining lifetime. Diet influences bone properties including bone metabolism, bone mineral density (BMD), bone geometry, microstructure, bone matrix mineralization and material level properties as well as muscle function which determines bone strength and fracture risk [1, 2] (Fig. 1). Some key relationships of diet to musculoskeletal health and bone fragility are depicted in Fig. 2.In addition to these dietary factors, appetite and the capacity to ingest the foods is important and of greater importance in the oldest old. Food seeking behavior is controlled by several hormonal pathways, as the example of UV light triggering food seeking behavior in males [3].

The aim of this article is to review and discuss recent observational studies and randomized controlled trials assessing the efficacy and safety of nutrients, of foods and of dietary patterns on bone health and fracture risk.

## Nutrients

### Minerals

Calcium and magnesium are two minerals that are dominant in bone mineral matrix and are often deficient in diets. Because circulating calcium and magnesium are maintained within a narrow range, their intakes are used as status indicators in the absence of good biochemical markers. Calcium intake varies considerably throughout the world [4]. To further complicate calcium status, older adults have decreased intestinal calcium absorption, further affected by frequent vitamin D deficiency. The relationship between dietary calcium intake and rates of bone loss and/or fracture risk in older postmenopausal women is unclear. Supplementation with calcium alone does not consistently reduce fracture risk [5]. In contrast, combining calcium and vitamin D lowers fracture risk by 5 to 15% and 13 to 30% for all and hip fractures, respectively, depending on the meta-analysis; the decrease is particularly detected in the frail elderly living in nursing homes (reviewed in [6]). Concern over increased cardiovascular risk with calcium supplementation led to decreased calcium supplementation, but the verification of this hypothesis has not been convincingly supported by clinical evidence [6–8]. The European Society for Clinical and Economic Aspects of Osteoporosis, Osteoarthritis and Musculoskeletal Diseases (ESCEO) and the International Foundation for Osteoporosis (IOF) recommend adequate calcium and vitamin D since most pivotal trials testing antiosteoporosis drugs with facture risk as primary outcome were conducted in calcium- and vitamin D-replete individuals [6].

Higher magnesium intake is associated with increased total hip and femoral neck BMD, with a positive association between hip BMD and magnesium intake (pooled beta coefficient: 0.03; 95%CI 0.01–0.06), but without any relationship with fracture risk [9, 10]. Magnesium and calcium have a common absorptive mechanism, and thus, can compete for absorption. High calcium and magnesium drinking water has also been associated with a lower risk of stroke in postmenopausal women [11].

### Protein

The association between BMD and dietary protein intake in adults has been studied in various systematic reviews and meta-analyses [12–16]. Protein intake accounted for 2 to 4% of BMD variance in one systematic review and meta-analysis of randomized controlled trials (RCT) [12]. In a meta-analysis of 5 RCTs, higher protein intake was associated with a 0.52% increase in lumbar spine BMD (95% CI: 0.06–0.97]) [14]. Among 3 intervention trials with protein intakes above the current Recommended Dietary Allowance (RDA) of 0.8 g/kg/day, one showed a + 1.9% difference versus controls in lumbar spine BMD with protein intakes at 163% of RDA for 26 weeks [15]. In a randomized, placebo controlled trial conducted in vitamin D and calcium replete subjects with a recent hip fracture, a protein supplement of 20 g per day for 6 months, led to a 50% reduction in proximal femur BMD loss at one year and to a shorter length of stay in a rehabilitation unit [17]. Using finite element analysis to estimate peripheral skeletal sites bone strength, a positive association was observed between predicted failure load and total, animal and dairy protein intake, but not with protein of vegetable origin [18–20].

Cohort studies show mixed results. One systematic review and meta-analysis of 4 cohort studies showed no significant reduction in hip fracture risk comparing the highest to the lowest quartile/quintile of dietary protein intakes (RR 0.75, 95% CI 0.47–1.20) [12]. Another review showed that higher dietary protein intake was associated with a 16% reduction of hip fracture risk compared to low protein, with a relative risk of 0.84 [0.73–0.95] [15]. In contrast, a review of 5 cohorts showed a relative risk of 0.89 [95% CI 0.82–0.97] [13]. Dietary protein at or above the current RDA could be beneficial for reducing hip fracture risk, but there have been no RCTs on dietary protein and fracture risk.

#### Dietary Protein and Calcium Interaction

There is evidence for a protein-calcium interaction. One of the first studies to support such an interaction was from a calcium-vitamin D intervention trial that showed a benefit of the intervention, but only in participants consuming the highest tertile of dietary protein [21]. Higher protein intake had no benefit in the placebo group; the benefit to femoral neck and whole body BMD was observed only in the calcium-vitamin D supplemented group. In middle-aged men and women, those with higher animal protein intake and a calcium intake of 800 mg/day or more reduced hip fracture risk (highest versus lowest tertile protein intake RR: 0.16; 95%CI 0.02–0.92), but had the opposite effect in those with lower calcium intake [22, 23]. A calcium-protein interaction on bone is not clear for plant proteins [18–20, 24].

#### Dietary Protein and the Acid–Base Theory

Protein excess has been a concern for increasing urinary calcium loss due to acidosis, and thereby, may cause bone loss. Recent studies have not supported this concept. In community-dwelling older men and women, no reduction of BMD nor increased fracture risk have been found in subjects with an acidic diet compared to those with neutral or alkaline diets [25–27]. In a longitudinal analysis of the Geneva Retirees cohort, acidic diet consumption was associated with attenuated age-related bone loss at the radius in women [27]. In contrast, in a longitudinal study carried out in a Mediterranean population at high cardiovascular risk, both low and high dietary acid intakes, as assessed by potential acid load or net endogenous acid production, were associated with an increased low trauma fracture risk in a U-shape relationship [28]. In view of the impaired protein assimilation of older individuals, the RDA of 0.8 g/kg body weight per day has been recommended to be increased to 1.2 g/kg per day in this age group, or even higher in cases of severe diseases [29]. Thus, insufficient dietary protein intake in the frail elderly is likely to be a more severe problem than protein excess.

### Beverages

Sugar-sweetened beverage consumption, particularly carbonated beverages, has been negatively associated with BMD [30]. This may be due to milk displacement [31]. Alternatively, sugar-sweetened beverages may have a direct negative effect through its association with higher fat [32].

Tea, particularly green tea, has also been associated with benefits to bone, hypothesized to be due to the flavonoids and polyphenols in tea.. In a Korean nationwide survey in postmenopausal women, the Odds Ratio for osteoporosis was 1.91 (95% CI: 1.13–3.23) in non-consumers (> 1 cup/day) compared to 1.82 (95% CI: 1.09–3.05) in consumers (> 1 cup green tea/day) [33]. Similarly, in a large Taiwan database of 42,742 subjects aged 45 to 74 years, with a median time follow-up of 8.5 years, multivariate adjusted HR for hip fracture was 0.69 (95% CI: 0.55–0.86) in the high tea consumption group as compared to tea abstainers [34]. In a UK population, a lower hip fracture risk of 36% (95% CI: 19–49) reduced risk in women with a BMI less than 18.5 kg/m^2^ was reported [35]. In the whole UK cohort however, and irrespective of BMI, hip fracture risk was lower by 4% (95% CI: 0–8) in consumers of both tea and coffee.

## Foods

### Dairy Products

Milk provides many nutrients needed for bone health. One liter of milk provides 32 to 35 g/l protein, 1,200 mg/l calcium, 930 mg/l phosphorus, essential trace elements and vitamins, and a number of cellular growth factors [36]. Dairy consumption was shown as early as 8′000 years BC by the presence of dairy proteins in dental calculus from skeletons in East Africa [37].

In adults, the effects of milk supplementation on bone health were assessed in a meta-analysis of 20 randomized controlled trials [38]. Compared to controls, milk supplementation resulted in a small but significant higher lumbar spine and total hip BMD (+ 0.004 and + 0.025 g/cm2, respectively), as well as with lower levels of CTX (-0.16 ng/ml), P1NP (-5.20 ng/ml) and PTH (-1.01 pg/ml). IGF-I was increased in the intervention groups (+ 1.79 nmol/l).

Observational studies show mixed results for the effect of dairy consumption and fracture risk (reviewed in [2, 36]). The dairy products’ matrix refers to the interaction and structure of its different components, the particular digestion, absorption and bioavailability of which may explain why various dairy products produce various effects. Fermented dairy products in particular have been shown protective against age-related bone loss [39, 40]. In a 20-year follow-up of 61,433 women, the risk of hip fracture was 0.70 and 0.64 for consumers of 400 g/day of yogurt or fermented milk, and of cheese, respectively [41]. For each serving (200 g of yogurt or 20 g of cheese), hip fracture risk was reduced by 10–15%. In a meta-analysis of 102,819 subjects, yoghurt consumption was associated with a 24% lower hip fracture risk [42]. Cheese consumption was shown to be protective against total fracture relative risk, i.e.0.90 (95% CI 0.86–0.95) compared to non-consumers in an umbrella review and meta-analysis of prospective studies [43].

Perhaps the strongest evidence for the role of dairy products on protection against fracture is from a large RCT in 7195 vitamin D-replete older (mean age 86 years) individuals living in nursing homes. The dairy group increased their calcium intake from 700 to 1142 mg/day and protein intake from 0.8 to 1.1 g/kg daily compared to the control group. This led to a reduction of 33% in all fractures, of 46% of hip fracture and of 10% of falls [44]. Mortality was not influenced.

A potential source of intolerance to cow milk is the presence of A1 beta-casein, in some cow breeds, particularly those of European origin, instead of A2 beta-casein, in Asian or African cattle [45]. Both beta-casein proteins, which represent 30% of total protein of cow milk, differ by only one nucleotide changing the codon in position 67 of the 209 amino acid protein, with a histidine replacing a proline. The digestion of A1 but not of A2 beta-casein produces beta-casomorphin-7, which activates µ-opioid receptors located along the gastro-intestinal tract and may explain an increase in gastro-intestinal transit time and occasional abdominal discomfort after milk consumption. However, there is no evidence that A1 beta-casein is harmful for human health [46].

The possible influence of dairy products particularly on cardiovascular health has been a contentious issue.. Newer evidence does not support a relationship between dairy consumption and risk of cardiovascular disease. In fact, in a large multinational cohort study of 136,384 individuals aged 35–70 years from 21 countries in 5 continents with a 9-year follow-up, dairy consumption was associated with lower risk of mortality and of major cardiovascular disease events [47]. In a systematic review and meta-analysis of prospective cohort studies, the relative risk for an increase of 1 serving/day of total dairies was 0.96 (95% CI: 0.94–0.97), 0.98 (95% CI: 0.95–1.0) and 0.96 (95% CI: 0.93–0.99) for hypertension, coronary heart disease and stroke, respectively [48]. Cheese consumption, in particular, was inversely associated with all-cause mortality, cardiovascular mortality, incident cardiovascular disease, coronary heart disease, stroke and even dementia in an umbrella review and meta-analysis including 186 observational studies [43].

### Fruits and Vegetables

Fruits and vegetables provide a variety of micronutrients, vitamins, phytochemicals with antioxidant properties, fiber, and an alkaline load. Fiber is the main source of prebiotics, which are non-digestible food components that pass undigested though the upper gastro-intestinal tract and stimulate the growth and/or activity of bacteria present in the large intestine by acting as substrate for them. Fermentation of fiber leads to the production of short chain fatty acids such as acetate, propionate, valerate, isovalerate, butyrate, and isobutyrate, which has been associated with gut health and improved mineral absorption [49, 50].

In elderly men and women, fruit and vegetable intake is associated with greater BMD [51, 52]. In men from the Framingham Offspring Study, femoral neck bone loss was higher in those with the lowest dietary fiber intake (first quartile) compared to the other quartiles (Q2, Q3, or Q4, with an annual BMD change of -0.15% compared to -0.009 to -0.03% (*p* < 0.04) [53]. In a dose–response observational study, less than 5 servings per day was associated with an exponential increase in hip fracture risk [54]. However, more than 5 servings per day of fruits and vegetables did not exert further protective effects. A meta-analysis including 5 large observational studies indicated a 8% reduction in hip fracture risk with regular fruits and vegetables intake [55]. In an umbrella review of observational studies including 330,417 subjects and 6,779 hip fractures, the relative risk of hip fractures was 0.81 (95% CI 0.68–0.96) when comparing the highest to the lowest vegetables consumption categories [56].

## Dietary Patterns

Increasingly, attention is focused on dietary patterns rather than individual nutrients or foods. Dietary patterns are defined as the quantity, proportion and combination of various foods, nutrients and drinks in diets, and their habitual frequency of consumption. Some dietary patterns have been shown to increase and others to decrease risk of fracture. The most stringent dietary pattern that avoids meat is a vegan diet. Strict adherence to a vegan diet leads to a variety of deficiencies [57] including low calcium intake and low vitamin D levels. Vegetarian and vegan patterns have been associated with lower bone mineral density and increased fracture risk [58]. A similar increase in fracture risk was found in vegan populations of either European or Asian origin. In a more recent study of 26,318 women enrolled in the UK Women’s Cohort study with 822 hip fractures, hip fracture risk was 1.33-fold higher (95% CI: 1.03–1.71) in vegetarians as compared with omnivores [59]. No decreased risk of fracture was observed in pescatarians (fish eaters) or occasional meat eaters. In a much larger cohort of UK men and women (7,638 vegetarians and 258,765 omnivores), the Hazard Ratio (HR) for hip fracture was 1.50 (95% CI: 1.18–1.91) [60]. Combining data in 76,000 individuals from the EPIC-Oxford and Oxford Vegetarian the HR for the risk of all fractures was 1.11 (95% CI: 1.02–1.21) in vegetarians as compared with meat-eaters and HR for risk of hip fracture 1.34 (95% CI: 1.12–1.61) [61]. In vegans HR was 1.50 (95% CI: 1.26–1.78) and 2.64 (95% CI: 1.90–3.67) for all typers of fractures and for those of the hip, respectively. These studies are summarized in Table 1.

**Table 1:** Risk of fracture in vegetarians and vegans compared to regular meat eaters

Author, year	Participants	Fracture risk	
		All fractures	Hip fracture
Ignacel, 2019 [58]	4 combined studies		
	Vegans (*n* = 5,690) vs regular meat eaters (*n* = 37,173)	RR: 1.44 (1.05–1.98)	NA
	5 combined studies		
	Vegetarians (*n* = 23,645) vs regular meat eaters (*n* = 42,658)	RR: 1.25 (0.92–1.71)	NA
Key, 2022 [61]	EPIC-Oxford (*n* = 65,000) + Oxford Vegetarian study (*n* = 11,000) men and women		
	Vegans vs regular meat eaters	HR: 1.50 (1.26–1.78); 1.43^#^	HR: 2.64 (1.90–3.67); 2.31^#^
	Vegetarians vs regular meat eaters	HR: 1.11 (1.02–1.21); 1.09^#^	HR: 1.34 (1.12–1.61); 1.25^#^
Webster, 2022 [59]	UK Women’s Cohort study (*n* = 26,318, 822 hip fractures)		
	Vegetarians (*n* = 4,393) vs regular meat eaters (*n* = 13,984)	NA	^*^HR: 1.33 (1.03–1.71)
Webster, 2023 [60]	Middle-aged UK Men and Women		
	Vegetarians (*n* = 7,638) vs regular meat eaters (*n* = 258,765)	NA	^*^HR: 1.50 (1.18–1.91)

*RR* Relative risk (95% CI); *HR* Hazard ratio (95% CI); *NA* Not available; ^#^ Adjusted for BMI; ^*^Multivariable-adjusted HR

Reprinted from [62] with permission from the publisher

In the Adventist Health Study 2, the HR for hip fracture depended on whether women were consuming calcium and vitamin D supplements; the HR was 2.99 (95% CI: 1.54–5.82) for vegan women compared to nonvegetarians not consuming these supplements but was substantially reduced if they were, i.e. 0.84 (95% CI: 0.42–1.66) [63]. In vegans, peripheral skeleton trabecular and cortical microstructure were altered compared with omnivores unless they practiced resistance training [64].

In contrast to the detriments to bone of dietary patterns that exclude animal protein, bone health is benefitted from a balanced diet like a Mediterranean diet [65–67]. In a meta-analysis of observational studies including 13,209 participants, total hip and trochanter BMD was positively associated with a greater adherence to a Mediterranean diet [65].

### Role of Gut Microbiota

At the beginning of the twentieth century, the Nobel Prize winner Yllia Metchnikoff suggested that health could be improved, senility delayed and longevity prolonged by modifying the gut microbiota through the ingestion of lactobacilli found in yogurt [68]. Evidence is mounting for a role of gut microbiota composition and function in bone and mineral homeostasis [69]. Adherence to a Mediterranean diet is associated with significant changes in the gut microbiota diversity, composition, and functions [70]. By 3 months on a Mediterranean diet, there was a marked increase in the short chain fatty acids propionate and butyrate production associated with an improvement in the intestinal barrier integrity in a cohort of 260 women [71]. An example of combining pre- and probiotics, as occurs in a Mediterranean diet, was associated with lower rates of hip fracture when comparing high to low consumers of fruits and vegetables and fermented milk [72].

A rather recent bioactive compound of interest is uroithin A. Urolithin A is a gut microbiota postbiotic derived from pomegranate juice. In a 4-month placebo-controlled randomized trial in middle-aged healthy adults, oral urolithin A supplementation improved muscle strength by about 12% [73]. Several preclinical studies have demonstrated an inhibition of osteoclastogenesis which should be studied further for the possible effect of urolithin A on bone health [74, 75].

## Conclusion

The aim of this review was to assess the role of nutritional intakes in osteoporosis and/or fracture prevention. Evidence suggests adequate calcium, vitamin D and protein are key nutritional strategies to reduce fracture risk. Fracture risk is increased in individuals under a diet devoid of dairy products, like a vegan diet. Dairy product consumption, particularly fermented dairy products, are associated with a lower risk of hip fracture. Fewer than 5 servings per day of fruits and vegetables is associated with higher hip fracture risk. Adherence to a Mediterranean diet, rich in fruits, vegetables, and dairy products reduces hip fracture risk. Such a dietary pattern also provides fibers, polyphenols, pre- and probiotics which influences gut microbiota composition and/or function. Thus, a balanced diet including minerals, protein, vitamin D, fruits and vegetables is an important strategy in the prevention of osteoporosis and fragility fracture.

## Key References

- Rizzoli R, Biver E, Brennan-Speranza TC (2021) Nutritional intake and bone health. Lancet Diabetes Endocrinol 9:606–621
  - Extensive review of the topic nutrition and bone health
- Iuliano S, Poon S, Robbins J, Bui M, Wang X, De Groot L, Van Loan M, Zadeh AG, Nguyen T, Seeman E (2021) Effect of dietary sources of calcium and protein on hip fractures and falls
  - The largest randomized trila assessing the effects of dairies on musculo-skeletal outcomes
- Chen Z, Ahmed M, Ha V, Jefferson K, Malik V, Ribeiro PAB, Zuchinali P, Drouin-Chartier JP (2022) Dairy Product Consumption and Cardiovascular Health: A Systematic Review and Meta-analysis of Prospective Cohort Studies. Adv Nutr 13:439–454
  - A reassuring review of the cardio-vascular risk in relation with dairy consumption

## Data Availability

No datasets were generated or analysed during the current study.

## References

[1] Rizzoli R. Postmenopausal osteoporosis: assessment and management. Best Pract Res Clin Endocrinol Metab. 2018;32:739–57. 30449552 10.1016/j.beem.2018.09.005

[2] Rizzoli R, Biver E, Brennan-Speranza TC. Nutritional intake and bone health. Lancet Diabetes Endocrinol. 2021;9:606–21. 34242583 10.1016/S2213-8587(21)00119-4

[3] Parikh S, Parikh R, Michael K, et al. Food-seeking behavior is triggered by skin ultraviolet exposure in males. Nat Metab. 2022;4:883–900. 35817855 10.1038/s42255-022-00587-9 PMC9314261

[4] Balk EM, Adam GP, Langberg VN, et al. Global dietary calcium intake among adults: a systematic review. Osteoporos Int. 2017;28:3315–24. 29026938 10.1007/s00198-017-4230-x PMC5684325

[5] Bolland MJ, Leung W, Tai V, Bastin S, Gamble GD, Grey A, Reid IR. Calcium intake and risk of fracture: systematic review. BMJ. 2015;351:h4580. 26420387 10.1136/bmj.h4580 PMC4784799

[6] Harvey NC, Biver E, Kaufman JM, et al. The role of calcium supplementation in healthy musculoskeletal ageing: an expert consensus meeting of the European Society for Clinical and Economic Aspects of Osteoporosis, Osteoarthritis and Musculoskeletal Diseases (ESCEO) and the International Foundation for Osteoporosis (IOF). Osteoporos Int. 2017;28:447–62. 27761590 10.1007/s00198-016-3773-6 PMC5274536

[7] Chung M, Tang AM, Fu Z, Wang DD, Newberry SJ. Calcium intake and cardiovascular disease risk: an updated systematic review and meta-analysis. Ann Intern Med. 2016;165:856–66. 27776363 10.7326/M16-1165

[8] Curtis EM, Cooper C, Harvey NC. Cardiovascular safety of calcium, magnesium and strontium: what does the evidence say? Aging Clin Exp Res. 2021;33:479–94. 33565045 10.1007/s40520-021-01799-x PMC7943433

[9] Farsinejad-Marj M, Saneei P, Esmaillzadeh A. Dietary magnesium intake, bone mineral density and risk of fracture: a systematic review and meta-analysis. Osteoporos Int. 2016;27:1389–99. 26556742 10.1007/s00198-015-3400-y

[10] Groenendijk I, van Delft M, Versloot P, van Loon LJC, de Groot L. Impact of magnesium on bone health in older adults: a systematic review and meta-analysis. Bone. 2022;154:116233. 34666201 10.1016/j.bone.2021.116233

[11] Helte E, Säve-Söderbergh M, Larsson SC, Åkesson A. Calcium and magnesium in drinking water and risk of myocardial infarction and stroke-a population-based cohort study. Am J Clin Nutr. 2022;116:1091–100. 35816459 10.1093/ajcn/nqac186 PMC9535516

[12] Darling AL, Millward DJ, Torgerson DJ, Hewitt CE, Lanham-New SA. Dietary protein and bone health: a systematic review and meta-analysis. Am J Clin Nutr. 2009;90:1674–92. 19889822 10.3945/ajcn.2009.27799

[13] Wu AM, Sun XL, Lv QB, Zhou Y, Xia DD, Xu HZ, Huang QS, Chi YL. The relationship between dietary protein consumption and risk of fracture: a subgroup and dose-response meta-analysis of prospective cohort studies. Sci Rep. 2015;5:9151. 25779888 10.1038/srep09151 PMC5376209

[14] Shams-White MM, Chung M, Du M, et al. Dietary protein and bone health: a systematic review and meta-analysis from the National Osteoporosis Foundation. Am J Clin Nutr. 2017;105:1528–43. 28404575 10.3945/ajcn.116.145110

[15] Wallace TC, Frankenfeld CL. Dietary protein intake above the current RDA and bone health: a systematic review and meta-analysis. J Am Coll Nutr. 2017;36:481–96. 28686536 10.1080/07315724.2017.1322924

[16] Darling AL, Manders RJF, Sahni S, Zhu K, Hewitt CE, Prince RL, Millward DJ, Lanham-New SA. Dietary protein and bone health across the life-course: an updated systematic review and meta-analysis over 40 years. Osteoporos Int. 2019;30:741–61. 30903209 10.1007/s00198-019-04933-8

[17] Schürch MA, Rizzoli R, Slosman D, Vadas L, Vergnaud P, Bonjour JP. Protein supplements increase serum insulin-like growth factor-I levels and attenuate proximal femur bone loss in patients with recent hip fracture. A randomized, double-blind, placebo-controlled trial. Ann Intern Med. 1998;128:801–9. 9599191 10.7326/0003-4819-128-10-199805150-00002

[18] Durosier-Izart C, Biver E, Merminod F, van Rietbergen B, Chevalley T, Herrmann FR, Ferrari SL, Rizzoli R. Peripheral skeleton bone strength is positively correlated with total and dairy protein intakes in healthy postmenopausal women. Am J Clin Nutr. 2017;105:513–25. 28077378 10.3945/ajcn.116.134676

[19] Langsetmo L, Shikany JM, Cawthon PM, Cauley JA, Taylor BC, Vo TN, Bauer DC, Orwoll ES, Schousboe JT, Ensrud KE. The association between protein intake by source and osteoporotic fracture in older men: a prospective cohort study. J Bone Miner Res. 2017;32:592–600. 27943394 10.1002/jbmr.3058 PMC5426558

[20] Groenendijk I, Grootswagers P, Santoro A, et al. Protein intake and bone mineral density: cross-sectional relationship and longitudinal effects in older adults. J Cachexia Sarcopenia Muscle. 2023;14:116–25. 36346154 10.1002/jcsm.13111 PMC9891984

[21] Dawson-Hughes B, Harris SS. Calcium intake influences the association of protein intake with rates of bone loss in elderly men and women. Am J Clin Nutr. 2002;75:773–9. 11916767 10.1093/ajcn/75.4.773

[22] Dargent-Molina P, Sabia S, Touvier M, Kesse E, Bréart G, Clavel-Chapelon F, Boutron-Ruault MC. Proteins, dietary acid load, and calcium and risk of postmenopausal fractures in the E3N French women prospective study. J Bone Miner Res. 2008;23:1915–22. 18665794 10.1359/jbmr.080712 PMC2929535

[23] Sahni S, Cupples LA, McLean RR, Tucker KL, Broe KE, Kiel DP, Hannan MT. Protective effect of high protein and calcium intake on the risk of hip fracture in the Framingham offspring cohort. J Bone Miner Res. 2010;25:2770–6. 20662074 10.1002/jbmr.194 PMC3179277

[24] Langsetmo L, Barr SI, Berger C, et al. Associations of protein intake and protein source with bone mineral density and fracture risk: a population-based cohort study. J Nutr Health Aging. 2015;19:861–8. 26412291 10.1007/s12603-015-0544-6 PMC5092173

[25] Pedone C, Napoli N, Pozzilli P, Lauretani F, Bandinelli S, Ferrucci L, Antonelli-Incalzi R. Quality of diet and potential renal acid load as risk factors for reduced bone density in elderly women. Bone. 2010;46:1063–7. 20005315 10.1016/j.bone.2009.11.031 PMC2881463

[26] Jia T, Byberg L, Lindholm B, Larsson TE, Lind L, Michaëlsson K, Carrero JJ. Dietary acid load, kidney function, osteoporosis, and risk of fractures in elderly men and women. Osteoporos Int. 2015;26:563–70. 25224295 10.1007/s00198-014-2888-x

[27] Papageorgiou M, Merminod F, Chevalley T, van Rietbergen B, Ferrari S, Rizzoli R, Biver E. Associations between age-related changes in bone microstructure and strength and dietary acid load in a cohort of community-dwelling, healthy men and postmenopausal women. Am J Clin Nutr. 2020;112:1120–31. 32678420 10.1093/ajcn/nqaa191

[28] García-Gavilán JF, Martínez A, Konieczna J, et al. U-shaped association between dietary acid load and risk of osteoporotic fractures in 2 populations at high cardiovascular risk. J Nutr. 2021;151:152–61. 33296471 10.1093/jn/nxaa335

[29] Bauer J, Biolo G, Cederholm T, et al. Evidence-based recommendations for optimal dietary protein intake in older people: a position paper from the PROT-AGE Study Group. J Am Med Dir Assoc. 2013;14:542–59. 23867520 10.1016/j.jamda.2013.05.021

[30] Ahn H, Park YK. Sugar-sweetened beverage consumption and bone health: a systematic review and meta-analysis. Nutr J. 2021;20:41. 33952276 10.1186/s12937-021-00698-1 PMC8101184

[31] Keller KL, Kirzner J, Pietrobelli A, St-Onge MP, Faith MS. Increased sweetened beverage intake is associated with reduced milk and calcium intake in 3- to 7-year-old children at multi-item laboratory lunches. J Am Diet Assoc. 2009;109:497–501. 19248869 10.1016/j.jada.2008.11.030 PMC2748414

[32] Bennett AM, Murray K, Ambrosini GL, Oddy WH, Walsh JP, Zhu K. Prospective associations of sugar-sweetened beverage consumption during adolescence with body composition and bone mass at early adulthood. J Nutr. 2022;152:399–407. 34791346 10.1093/jn/nxab389 PMC8826835

[33] Lee DB, Song HJ, Paek YJ, Park KH, Seo YG, Noh HM. Relationship between regular green tea intake and osteoporosis in korean postmenopausal women: a nationwide study. Nutrients. 2021;14:87. 10.3390/nu14010087 PMC8746552 35010962

[34] Huang YP, Chen LS, Feng SH, Liang YS, Pan SL. Tea consumption and the risks of osteoporosis and hip fracture: a population-based longitudinal follow-up study. Osteoporos Int. 2023;34:101–9. 36241848 10.1007/s00198-022-06569-7 PMC9813189

[35] Webster J, Greenwood DC, Cade JE. Foods, nutrients and hip fracture risk: a prospective study of middle-aged women. Clin Nutr. 2022;41:2825–32. 36402009 10.1016/j.clnu.2022.11.008

[36] Rizzoli R. Dairy products and bone health. Aging Clin Exp Res. 2022;34:9–24. 34494238 10.1007/s40520-021-01970-4 PMC8794967

[37] Bleasdale M, Richter KK, Janzen A, et al. Ancient proteins provide evidence of dairy consumption in eastern Africa. Nat Commun. 2021;12:632. 33504791 10.1038/s41467-020-20682-3 PMC7841170

[38] Hidayat K, Chen JS, Wang TC, Liu YJ, Shi YJ, Su HW, Liu B, Qin LQ. The effects of milk supplementation on bone health indices in adults: a meta-analysis of randomized controlled trials. Adv Nutr. 2022;13:1186–99. 34792092 10.1093/advances/nmab136 PMC9340984

[39] Rizzoli R, Biver E. Effects of fermented milk products on bone. Calcif Tissue Int. 2018;102:489–500. 28823001 10.1007/s00223-017-0317-9

[40] Biver E, Durosier-Izart C, Merminod F, Chevalley T, van Rietbergen B, Ferrari SL, Rizzoli R. Fermented dairy products consumption is associated with attenuated cortical bone loss independently of total calcium, protein, and energy intakes in healthy postmenopausal women. Osteoporos Int. 2018;29:1771–82. 29725715 10.1007/s00198-018-4535-4

[41] Michaëlsson K, Wolk A, Langenskiöld S, Basu S, Warensjö Lemming E, Melhus H, Byberg L. Milk intake and risk of mortality and fractures in women and men: cohort studies. BMJ. 2014;349:g6015. 25352269 10.1136/bmj.g6015 PMC4212225

[42] Ong AM, Kang K, Weiler HA, Morin SN. Fermented milk products and bone health in postmenopausal women: a systematic review of randomized controlled trials, prospective cohorts, and case-control studies. Adv Nutr. 2020;11:251–65. 31603185 10.1093/advances/nmz108 PMC7442363

[43] Zhang M, Dong X, Huang Z, Li X, Zhao Y, Wang Y, Zhu H, Fang A, Giovannucci EL. Cheese consumption and multiple health outcomes: an umbrella review and updated meta-analysis of prospective studies. Adv Nutr. 2023;14:1170–86. 37328108 10.1016/j.advnut.2023.06.007 PMC10509445

[44] Iuliano S, Poon S, Robbins J, Bui M, Wang X, De Groot L, Van Loan M, Zadeh AG, Nguyen T, Seeman E. Effect of dietary sources of calcium and protein on hip fractures and falls in older adults in residential care: cluster randomised controlled trial. BMJ. 2021;375:n2364. 34670754 10.1136/bmj.n2364 PMC8527562

[45] Kay SS, Delgado S, Mittal J, Eshraghi RS, Mittal R, Eshraghi AA. Beneficial effects of milk having A2 β-casein protein: myth or reality? J Nutr. 2021;151:1061–72. 33693747 10.1093/jn/nxaa454

[46] Giribaldi M, Lamberti C, Cirrincione S, Giuffrida MG, Cavallarin L. A2 milk and BCM-7 peptide as emerging parameters of milk quality. Front Nutr. 2022;9:842375. 35571904 10.3389/fnut.2022.842375 PMC9094626

[47] Dehghan M, Mente A, Rangarajan S, et al. Association of dairy intake with cardiovascular disease and mortality in 21 countries from five continents (PURE): a prospective cohort study. Lancet. 2018;392:2288–97. 30217460 10.1016/S0140-6736(18)31812-9

[48] Chen Z, Ahmed M, Ha V, Jefferson K, Malik V, Ribeiro PAB, Zuchinali P, Drouin-Chartier JP. Dairy product consumption and cardiovascular health: a systematic review and meta-analysis of prospective cohort studies. Adv Nutr. 2022;13:439–54. 34550320 10.1093/advances/nmab118 PMC8970833

[49] Weaver CM. Diet, gut microbiome, and bone health. Curr Osteoporos Rep. 2015;13:125–30. 25616772 10.1007/s11914-015-0257-0 PMC4996260

[50] Whisner CM, Castillo LF. Prebiotics, bone and mineral metabolism. Calcif Tissue Int. 2018;102:443–79. 29079996 10.1007/s00223-017-0339-3 PMC5851694

[51] Tucker KL, Hannan MT, Chen H, Cupples LA, Wilson PW, Kiel DP. Potassium, magnesium, and fruit and vegetable intakes are associated with greater bone mineral density in elderly men and women. Am J Clin Nutr. 1999;69:727–36. 10197575 10.1093/ajcn/69.4.727

[52] Qiu R, Cao WT, Tian HY, He J, Chen GD, Chen YM. Greater intake of fruit and vegetables is associated with greater bone mineral density and lower osteoporosis risk in middle-aged and elderly adults. PLoS ONE. 2017;12:e0168906. 28045945 10.1371/journal.pone.0168906 PMC5207626

[53] Dai Z, Zhang Y, Lu N, Felson DT, Kiel DP, Sahni S. Association between dietary fiber intake and bone loss in the Framingham offspring study. J Bone Miner Res. 2018;33:241–9. 29024045 10.1002/jbmr.3308 PMC5990003

[54] Byberg L, Bellavia A, Orsini N, Wolk A, Michaëlsson K. Fruit and vegetable intake and risk of hip fracture: a cohort study of Swedish men and women. J Bone Miner Res. 2015;30:976–84. 25294687 10.1002/jbmr.2384

[55] Brondani JE, Comim FV, Flores LM, Martini LA, Premaor MO. Fruit and vegetable intake and bones: a systematic review and meta-analysis. PLoS ONE. 2019;14:e0217223. 31150426 10.1371/journal.pone.0217223 PMC6544223

[56] Angelino D, Godos J, Ghelfi F, et al. Fruit and vegetable consumption and health outcomes: an umbrella review of observational studies. Int J Food Sci Nutr. 2019;70:652–67. 30764679 10.1080/09637486.2019.1571021

[57] O’Keefe JH, O’Keefe EL, Lavie CJ, Cordain L. Debunking the vegan myth: the case for a plant-forward omnivorous whole-foods diet. Prog Cardiovasc Dis. 2022;74:2–8. 35944662 10.1016/j.pcad.2022.08.001

[58] Iguacel I, Miguel-Berges ML, Gómez-Bruton A, Moreno LA, Julián C. Veganism, vegetarianism, bone mineral density, and fracture risk: a systematic review and meta-analysis. Nutr Rev. 2019;77:1–18. 30376075 10.1093/nutrit/nuy045

[59] Webster J, Greenwood DC, Cade JE. Risk of hip fracture in meat-eaters, pescatarians, and vegetarians: results from the UK Women’s Cohort Study. BMC Med. 2022;20:275. 35948956 10.1186/s12916-022-02468-0 PMC9367078

[60] Webster J, Greenwood DC, Cade JE. Risk of hip fracture in meat-eaters, pescatarians, and vegetarians: a prospective cohort study of 413,914 UK Biobank participants. BMC Med. 2023;21:278. 37501206 10.1186/s12916-023-02993-6 PMC10375740

[61] Key TJ, Papier K, Tong TYN. Plant-based diets and long-term health: findings from the EPIC-Oxford study. Proc Nutr Soc. 2022;81:190–8. 35934687 10.1017/S0029665121003748 PMC7613518

[62] Rizzoli R, Chevalley T. Bone health: biology and nutrition. Curr Opin Clin Nutr Metab Care. 2024;27:24–30. 37922025 10.1097/MCO.0000000000000988 PMC10720787

[63] Thorpe DL, Beeson WL, Knutsen R, Fraser GE, Knutsen SF. Dietary patterns and hip fracture in the Adventist Health Study 2: combined vitamin D and calcium supplementation mitigate increased hip fracture risk among vegans. Am J Clin Nutr. 2021;114:488–95. 33964850 10.1093/ajcn/nqab095 PMC8435998

[64] Wakolbinger-Habel R, Reinweber M, König J, Pokan R, König D, Pietschmann P, Muschitz C. Self-reported resistance training is associated with better HR-pQCT-derived bone microarchitecture in vegan people. J Clin Endocrinol Metab. 2022;107:2900–11. 35924941 10.1210/clinem/dgac445

[65] Noori M, Jayedi A, Khan TA, Moradi S, Shab-Bidar S. Mediterranean dietary pattern and bone mineral density: a systematic review and dose-response meta-analysis of observational studies. Eur J Clin Nutr. 2022;76:1657–64. 35173291 10.1038/s41430-022-01093-7

[66] Malmir H, Saneei P, Larijani B, Esmaillzadeh A. Adherence to Mediterranean diet in relation to bone mineral density and risk of fracture: a systematic review and meta-analysis of observational studies. Eur J Nutr. 2018;57:2147–60. 28638994 10.1007/s00394-017-1490-3

[67] Andreo-López MC, Contreras-Bolívar V, García-Fontana B, García-Fontana C, Muñoz-Torres M. The influence of the mediterranean dietary pattern on osteoporosis and sarcopenia. Nutrients. 2023;15:3224. 10.3390/nu15143224 PMC10385532 37513646

[68] Mackowiak PA. Recycling metchnikoff: probiotics, the intestinal microbiome and the quest for long life. Front Public Health. 2013;1:52. 24350221 10.3389/fpubh.2013.00052 PMC3859987

[69] Rizzoli R. Nutritional influence on bone: role of gut microbiota. Aging Clin Exp Res. 2019;31:743–51. 30710248 10.1007/s40520-019-01131-8

[70] Khavandegar A, Heidarzadeh A, Angoorani P, Hasani-Ranjbar S, Ejtahed HS, Larijani B, Qorbani M. Adherence to the Mediterranean diet can beneficially affect the gut microbiota composition: a systematic review. BMC Med Genomics. 2024;17:91. 38632620 10.1186/s12920-024-01861-3 PMC11022496

[71] Seethaler B, Nguyen NK, Basrai M, Kiechle M, Walter J, Delzenne NM, Bischoff SC. Short-chain fatty acids are key mediators of the favorable effects of the Mediterranean diet on intestinal barrier integrity: data from the randomized controlled LIBRE trial. Am J Clin Nutr. 2022;116:928–42. 36055959 10.1093/ajcn/nqac175

[72] Michaëlsson K, Wolk A, Lemming EW, Melhus H, Byberg L. Intake of milk or fermented milk combined with fruit and vegetable consumption in relation to hip fracture rates: a cohort study of Swedish women. J Bone Miner Res. 2018;33:449–57. 29083056 10.1002/jbmr.3324

[73] Singh A, D’Amico D, Andreux PA, Fouassier AM, Blanco-Bose W, Evans M, Aebischer P, Auwerx J, Rinsch C. Urolithin A improves muscle strength, exercise performance, and biomarkers of mitochondrial health in a randomized trial in middle-aged adults. Cell Rep Med. 2022;3:100633. 35584623 10.1016/j.xcrm.2022.100633 PMC9133463

[74] Tao H, Tao Y, Yang C, et al. Gut metabolite urolithin A inhibits osteoclastogenesis and senile osteoporosis by enhancing the autophagy capacity of bone marrow macrophages. Front Pharmacol. 2022;13:875611. 35645801 10.3389/fphar.2022.875611 PMC9135380

[75] Wei W, Peng C, Gu R, Yan X, Ye J, Xu Z, Sheng X, Huang G, Guo Y. Urolithin A attenuates RANKL-induced osteoclastogenesis by co-regulating the p38 MAPK and Nrf2 signaling pathway. Eur J Pharmacol. 2022;921:174865. 35231470 10.1016/j.ejphar.2022.174865

